# Quantitative Total-Body Imaging of Blood Flow with High-Temporal-Resolution Early Dynamic ^18^F-FDG PET Kinetic Modeling

**DOI:** 10.2967/jnumed.124.268706

**Published:** 2025-06

**Authors:** Kevin J. Chung, Abhijit J. Chaudhari, Lorenzo Nardo, Terry Jones, Moon S. Chen, Ramsey D. Badawi, Simon R. Cherry, Guobao Wang

**Affiliations:** 1 Department of Radiology, University of California Davis Health, Sacramento, California;; 2 Department of Internal Medicine, University of California Davis Health, Sacramento, California; and; 3 Department of Biomedical Engineering, University of California at Davis, Davis, California

**Keywords:** total-body PET, blood-flow/perfusion imaging, high-temporal resolution dynamic imaging, tracer kinetic modeling, distributed kinetic modeling

## Abstract

Past efforts to measure blood flow with the widely available radiotracer ^18^F-FDG were limited to tissues with high ^18^F-FDG extraction fraction. In this study, we developed an early dynamic ^18^F-FDG PET method with high-temporal-resolution (HTR) kinetic modeling to assess total-body blood flow based on deriving the vascular phase of ^18^F-FDG transit and conducted a pilot comparison study against a ^11^C-butanol flow-tracer reference. **Methods:** The first 2 min of dynamic PET scans were reconstructed at HTR (60 × 1 s/frame, 30 × 2 s/frame) to resolve the rapid passage of the radiotracer through blood vessels. In contrast to existing methods that use blood-to-tissue transport rate as a surrogate of blood flow, our method directly estimated blood flow using a distributed kinetic model (adiabatic approximation to tissue homogeneity [AATH] model). To validate our ^18^F-FDG measurements of blood flow against a reference flow-specific radiotracer, we analyzed total-body dynamic PET images of 6 human participants scanned with both ^18^F-FDG and ^11^C-butanol. An additional 34 total-body dynamic ^18^F-FDG PET images of healthy participants were analyzed for comparison against published blood-flow ranges. Regional blood flow was estimated across the body, and total-body parametric imaging of blood flow was conducted for visual assessment. AATH and standard compartment model fitting was compared using the Akaike information criterion at different temporal resolutions. **Results:** ^18^F-FDG blood flow was in quantitative agreement with flow measured from ^11^C-butanol across same-subject regional measurements (Pearson correlation coefficient, 0.955; *P* < 0.001; linear regression slope and intercept, 0.973 and –0.012, respectively), which was visually corroborated by total-body blood-flow parametric imaging. Our method resolved a wide range of blood-flow values across the body in broad agreement with published ranges (e.g., healthy cohort values of 0.51 ± 0.12 mL/min/cm^3^ in the cerebral cortex and 2.03 ± 0.64 mL/min/cm^3^ in the lungs). HTR (1–2 s/frame) was required for AATH modeling. **Conclusion:** Total-body blood-flow imaging was feasible using early dynamic ^18^F-FDG PET with HTR kinetic modeling. This method may be combined with standard ^18^F-FDG PET methods to enable efficient single-tracer multiparametric flow-metabolism imaging, with numerous research and clinical applications in oncology, cardiovascular disease, pain medicine, and neuroscience.

## Visual Abstract

**Figure:**
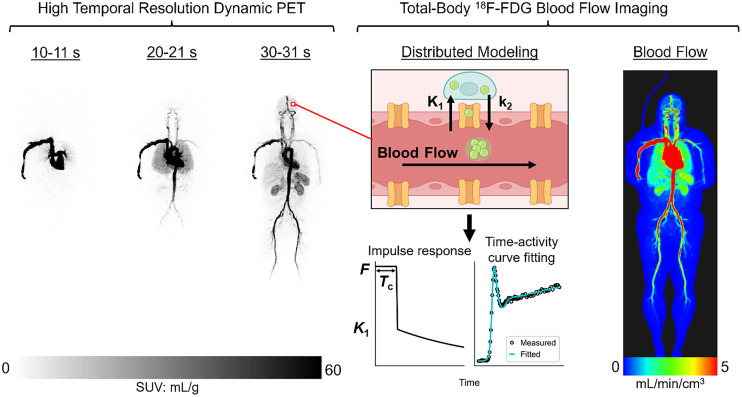

Imaging blood flow has garnered considerable interest over the past 50 y, as its dysfunction is characteristic in many diseases (1–3). PET imaging with a blood-flow–specific radiotracer, such as ^11^C-butanol or ^15^O-water, is widely considered the gold standard for blood-flow imaging (4–6). These flow radiotracers are freely diffusible across capillary membranes (4–6); accordingly, the measured PET signal is closely proportional to blood flow. Blood flow can then be quantified by a standard 1-tissue compartment (S1TC) model because of the complete or near-complete extraction of these freely diffusible flow radiotracers (4–6). Importantly, these flow radiotracers are highly extracted in tissue across the entire body and allow total-body imaging of blood flow (4,5,7).

However, the short half-lives of the radioisotopes in flow radiotracers create practical challenges that hinder their broader accessibility. ^15^O-water has a half-life of 2.04 min, which necessitates an on-site cyclotron and a dose-delivery system (8). ^11^C-butanol has a longer half-life (20.40 min) but still requires nearby production, thus limiting its access to urban or research PET centers. Other flow radiotracers, such as ^82^RbCl and ^13^N-ammonia, similarly have short radioisotope half-lives and high costs in addition to nonlinear uptake with flow (9). A blood-flow imaging method using a widely available radiotracer, such as ^18^F-FDG, may mitigate these challenges and open opportunities for imaging of blood flow and glucose metabolism with a single-tracer dynamic scan.

Early dynamic ^18^F-FDG PET has been used to measure blood flow in select tissues, such as tumors (10), liver (11), and myocardium (12), where ^18^F-FDG is moderately to highly extracted. The first 2- to 3-min dynamic ^18^F-FDG PET signal is principally weighted toward the initial delivery of the radiotracer to the tissue (13), and the higher regional extraction fraction makes the analysis amenable to simplified modeling, like that of freely diffusible flow radiotracers. However, these approaches are not generally applicable to other regions, such as the brain, with lower ^18^F-FDG extraction fractions (14,15).

An intravenously injected tracer is delivered to local tissue vasculature at a rate equal to blood flow. Standard compartmental models neglect this transient process, but distributed kinetic models explicitly model the blood flow and transit time associated with the radiotracer traversing the blood vessels (16,17). Although described several decades ago, distributed kinetic models had limited application in PET due to the poor temporal resolution and statistical quality of time–activity curves measured with conventional PET scanners (18,19).

Total-body PET has substantially greater sensitivity (20–22) compared with conventional PET systems and allows high-temporal resolution (HTR) dynamic imaging (21,23) and kinetic modeling (13,24,25). This may revitalize opportunities to apply distributed kinetic models for blood-flow estimation with ^18^F-FDG in various tissues. Here, we describe the development of an early dynamic ^18^F-FDG PET method for total-body blood-flow imaging with HTR kinetic modeling and its validation against an ^11^C-butanol reference in a subset of participants scanned with both radiotracers.

## MATERIALS AND METHODS

### Total-Body Dynamic PET

Two human cohorts were pooled in this study, each separately approved by the institutional review board at the University of California, Davis. Written informed consent was obtained for all participants. All participants received total-body dynamic imaging on the uEXPLORER PET/CT system (United Imaging Health Care), with the scan commencing immediately before bolus injection of the radiotracer.

The first cohort comprised 6 participants (4 women; mean age, 67 ± 15 y) with chronic low-back myofascial pain who underwent total-body dynamic PET, receiving bolus injections of both ^18^F-FDG (98 ± 9 MBq) and ^11^C-butanol PET (268 ± 6 MBq) at 2 scanning sessions within 14 d (Clinicaltrials.gov identifier NCT05876858). The median interval between scans was 9 d (range, 0–14 d). Two participants were scanned on the same day with ^11^C-butanol scanning commencing first, followed by an interval of at least 3 h before the ^18^F-FDG PET to allow ^11^C to decay to negligible levels. The second cohort comprised 34 healthy participants (21 women; mean age, 51 ± 13 y) with no self-reported history of cancer or myocardial infarction in the past 5 y (26). Participants were scanned with total-body dynamic ^18^F-FDG PET (mean injected activity, 358 ± 33 MBq, bolus injection), and their data were used for methodologic development and validation against literature blood-flow ranges. Two participants in the first cohort and 20 participants in the second cohort self-identified as belonging to racial or ethnic minorities (26).

For all dynamic scans, the first 2 min were reconstructed at HTR (60 × 1 s, 30 × 2 s) using reconstruction software provided by the vendor. This involved a time-of-flight ordered-subset expectation-maximum algorithm-based reconstruction without point-spread function modeling and with 4 iterations, 20 subsets, and standard corrections for attenuation, scatter, randoms, dead time, and decay (22). We used a matrix size of 150 × 150 × 486 and an isotropic voxel size of 4 mm.

### Tracer Kinetic Modeling of Blood Flow from Dynamic ^18^F-FDG Data

Existing methods to measure blood flow with ^18^F-FDG have been limited to select tissue with high extraction fraction such that the blood-to-tissue transport rate (*K*_1_) approximates blood flow directly (10,11) or by nonlinear calibration (12). *K*_1_ is defined as the product of blood flow (*F*) and extraction fraction (*E*):

$$K_{1}=FE.$$ Eq. 1

Equation 1 shows that *K*_1_ is a good approximation of blood flow only when *E* is close to 1.

**FIGURE 1.**
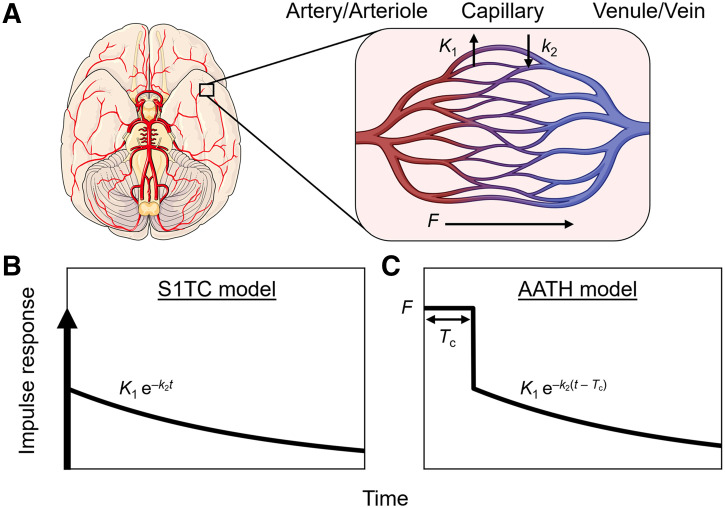
Modeling intravascular delivery of PET tracers. (A) PET voxel partly comprises arteries, arterioles, capillaries, venules, and veins. PET tracers are initially delivered to and circulate through these vascular volumes via blood flow (*F*). Tracer transport from blood into (*K*_1_) and out of tissue (*k*_2_) occurs almost exclusively at capillary level. S1TC model (B) assumes that tracer instantaneously mixes in vascular volume, and effectively mean vascular transit time (*T_c_*) is zero. AATH model (C) accounts for plug flow with single transit time (*T_c_*) for tracer to traverse total vascular volume via blood flow.

^18^F-FDG *K*_1_ can be measured with early dynamic imaging and an S1TC model, as the phosphorylation and dephosphorylation of ^18^F-FDG is not identifiable during the first few minutes of the dynamic scan (13,27). The impulse response function, $R^{S1TC}(t)$, of the S1TC is determined using Equation 2.

$$R^{S1TC}(t)=\left\{\begin{array}{cc} v_{b} & t=0,\\ K_{1}e^{-k_{2}t} & t>0, \end{array}\right.$$ Eq. 2

where $v_{b}$ is the blood volume and $K_{1}$ and $k_{2}$ are the blood-to-tissue transport and clearance rates, respectively (Fig. 1). Here, the value of $v_{b}$ at t=0 reflects the compartmental assumption that radiotracer instantaneously and uniformly mixes in regional blood vessels.

In reality, the radiotracer requires a nonzero transit time to traverse the length of the blood vessels at a rate equal to blood flow. This can be explicitly modeled in distributed parameter models (16,17). Here, we used the adiabatic approximation to tissue homogeneity (AATH) model (17), a distributed kinetic model with a closed-form time-domain solution that explicitly models intravascular blood flow and a mean vascular transit time. The impulse response function, $R^{\mathrm{AATH}}(t)$ can be determined using Equation 3.

$$R^{\mathrm{AATH}}\left(t\right)=\left\{\begin{array}{cc} F & 0\leq t<T_{c}\\ K_{1}e^{-k_{2}\left(t-T_{c}\right)} & t\geq T_{c} \end{array}\right.,$$ Eq. 3

where *F* is intravascular blood flow and $T_{c}$ is the mean vascular transit time for the radiotracer to pass through the total vascular blood volume ($v_{b)}$ in a voxel. $T_{c}$ is equal to the ratio of the blood volume to blood flow ($T_{c}=v_{b}/F$) (Fig. 1). Here, *F* is modeled separately from $K_{1}$. This method differs from conventional methods using extraction fraction correction (12) of $K_{1}$ to approximate blood flow.

$R^{\mathrm{AATH}}\left(t\right)$ describes a finite-time vascular phase ($0\leq t<T_{c}$) and a tissue phase ($t\geq T_{c}$). During the vascular phase, the radiotracer traverses the total vascular blood volume (arteries, arterioles, capillaries, venules, and veins) residing in a voxel at a uniform rate equal to the blood flow (plug flow). Capillary transit occurs for a fraction of this time during which a fraction (*E*) of the radiotracer is extracted to the extravascular tissue compartment. At the start of the tissue phase ($t=T_{c}$), the unextracted fraction (1 – *E*) is removed from the voxel volume through the venous outlet by blood flow. The extracted radiotracer then returns from the extravascular tissue compartment to the intravascular space and is cleared through the venous outlet by blood flow ($t>T_{c}$). Accordingly, the tissue phase of $R^{\mathrm{AATH}}\left(t\right)$ follows an exponential decay, like the S1TC model, with their response functions mainly differing by the presence of a nonzero-length vascular phase in the AATH model. We expect that the AATH and S1TC fittings may perform similarly at high extraction fractions as blood flow becomes similar to *K*_1_.

For a general arterial input, $C_{a}\left(t\right)$, the tissue time–activity curve, Q(t), can be derived using Equation 4.

$$Q\left(t\right)=C_{a}\left(t-t_{d}\right)⊗R\left(t\right),$$ Eq. 4

where $t_{d}$ is the time delay between radiotracer arrival at the measured arterial input location and local tissue vasculature. We used a basis function method with time-delay correction for least-squares parameter estimation using parametric forms of each model as described previously (28,29) and detailed in the supplemental materials, available at http://jnm.snmjournals.org (30–33). The AATH model was applied for both ^18^F-FDG and ^11^C-butanol.

### Image Analysis

Total-body PET enabled noninvasive measurement of an image-derived input function for kinetic analysis. The ascending aorta was used for kinetic modeling of all tissue except the lungs, for which a right ventricle input function was used (24,34,35). Early ^18^F-FDG kinetics were quantified by analyzing regional time–activity curves obtained from tissue segmentations in 10 regions of interest (supplemental materials) (36–40).

Total-body parametric images of early kinetics were generated by voxelwise kinetic modeling on 4-mm isotropic reconstructions. The dynamic and parametric images were smoothed by the kernel method, which is analogous to nonlocal means denoising (41,42). Prior composite images were derived from multiple static PET images (^18^F-FDG, 0–5, 5–20, 20–40, and 40–60 min; ^11^C-butanol, 0–1, 1–2, and 2–3 min), using 49 nearest neighbors within a 9 × 9 × 9 voxel neighborhood, as in our previous work (41,42).

### Evaluating Time–Activity Curve Fitting

We compared the quality of the AATH and S1TC model time–activity curve fits using the Akaike information criterion (AIC) (supplemental materials) (43). The difference in AIC (AATH minus S1TC) was computed for each region of interest. A lower AIC indicated better fitting after accounting for the number of model parameters and the residual fitting error. Practical identifiability analysis was also performed as in previous work (27) to determine the reliability of AATH parameter estimates.

To evaluate the effect of temporal resolution on the suitability of the AATH model, we frame-averaged each measured regional time–activity curve in the healthy ^18^F-FDG PET cohort at 1-, 2-, 3-, 5-, and 10-s/frame intervals. The resampled data were fitted with the AATH and S1TC models, and AIC differences were compared for each region and frame interval.

### Validating ^18^F-FDG Blood-Flow Quantification

The mean and SD of regional blood-flow values estimated with the AATH model were computed for all participants. In participants with both ^18^F-FDG and ^11^C-butanol PET images, we performed correlation and Bland–Altman analyses (44) of regional blood-flow estimates between radiotracers. Total-body blood-flow parametric images were visually compared between radiotracers. For the healthy ^18^F-FDG PET cohort, we compared their average regional values against published ranges (Supplemental Table 1) (4,6,18,45–66).

## RESULTS

### Time–Activity Curve Fitting and Model Selection

**FIGURE 2.**
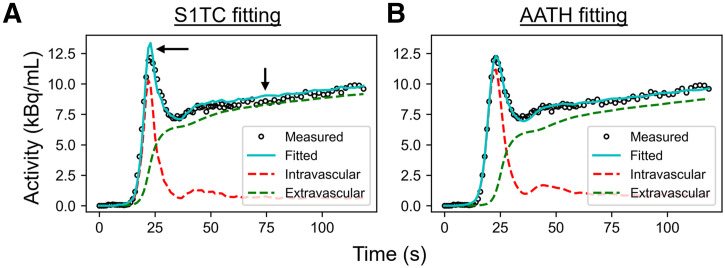
Time–activity curve fits in cortical gray matter using S1TC model (A) and AATH model (B) at HTR (60 × 1 s/frame, 30 × 2 s/frame). Dashed red and green lines represent intravascular and extravascular components of fitted curve, respectively. Black arrows indicate areas where S1TC fitting was poor.

**FIGURE 3.**
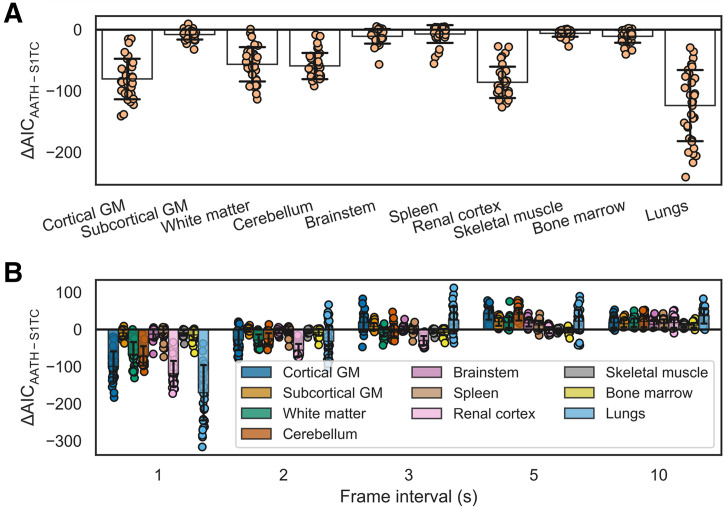
Difference in AIC between AATH and S1TC models using original HTR data (60 × 1 s/frame, 30 × 2 s/frame) (A) and at different simulated frame intervals (B). Negative AIC indicates preference toward AATH model. GM = gray matter.

An example HTR ^18^F-FDG time–activity curve fitting in the cortical gray matter with the S1TC and AATH models is shown in Figure 2. The first-pass peak, which was accurately measured with HTR dynamic imaging, was better fitted with the AATH model compared with the S1TC model. Furthermore, the peak of the intravascular component (dashed red line) of the AATH-fitted curve better aligned with the peak of the measured curve. The intravascular component is a scaled version of the arterial input function (Supplemental Eq. 4) in the S1TC model fit, whereas it is a smoothed, widened version in the AATH fit due to the nonzero *T_c_* (Supplemental Eq. 2). Accordingly, the intravascular distribution of the S1TC-fitted curve was smaller than that with the AATH model fit; to compensate, the extravascular distribution of the S1TC-fitted curve grew larger than that of the AATH model fit. In all regions of interest investigated, the AATH model was preferred on average over the S1TC model across 34 HTR dynamic ^18^F-FDG scans of healthy participants (Fig. 3A). Similarly, for ^11^C-butanol, S1TC model fitting was worse on average (Supplemental Fig. 1), and this justified our use of the AATH model for blood-flow comparisons against ^18^F-FDG.

Figure 3B illustrates the difference in AIC between the AATH and S1TC models at different temporal resolutions and in different tissue regions for the ^18^F-FDG cohort of 34 healthy participants. Using the AIC, the AATH model had improved fitting over the S1TC model at 1- to 2-s/frame intervals, though the average magnitude of AIC differences was lower at 2 s/frame. Beyond 3-s/frame intervals, the S1TC model was clearly preferred by the AIC.

### Validation of ^18^F-FDG PET Blood Flow Against ^11^C-Butanol PET

**FIGURE 4.**
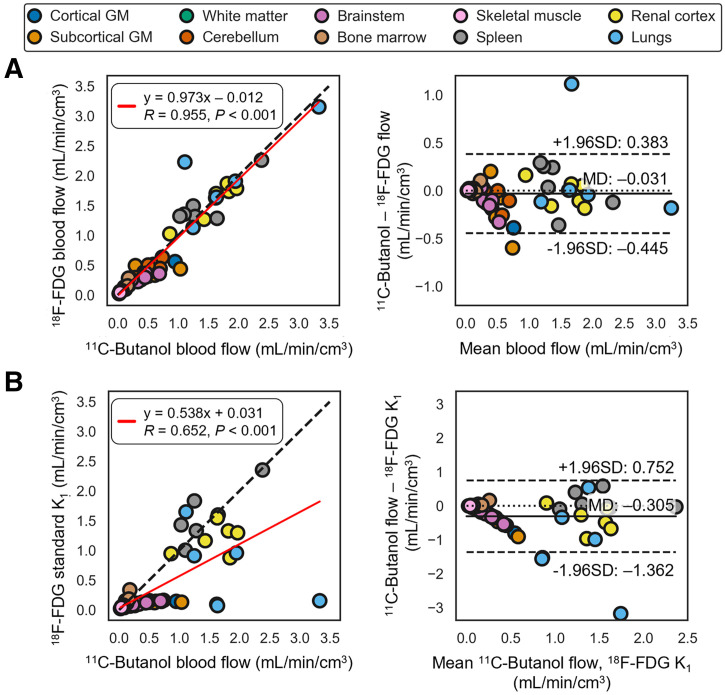
Correlation (left) and Bland–Altman (right) plots comparing ^11^C-butanol blood flow against ^18^F-FDG blood flow with AATH model (A) and S1TC model (B) *K*_1_ in same participants. GM = gray matter; MD = mean difference.

^11^C-butanol and ^18^F-FDG blood flow were compared across all 6 participants, each with 10 tissue regions, using correlation and Bland–Altman analyses (Figs. 4A and 4B). ^18^F-FDG blood flow estimated with our proposed method had strong quantitative agreement with the ^11^C-butanol reference measurement, with a Pearson correlation coefficient of 0.955 (*P* < 0.001) and a linear regression slope and intercept of 0.973 and –0.012, respectively. The mean difference in blood flow (^18^F-FDG minus ^11^C-butanol) was –0.031 mL/min/cm^3^, indicating that our ^18^F-FDG blood-flow measures marginally underestimated that of ^11^C-butanol, on average. The Bland–Altman 95% limits of agreement were –0.445 to 0.383 mL/min/cm^3^, with the larger differences mainly driven by tissues with higher blood flow. One participant had severe intraframe respiratory motion during the ^11^C-butanol scan, which prevented accurate lung blood-flow quantification and substantial overestimation (>1.0 mL/min/cm^3^) with our ^18^F-FDG PET method. Further analyses stratified by regions with similar blood flow are shown in Supplemental Figures 2 and 3. In general, S1TC ^18^F-FDG *K*_1_ did not strongly agree with ^11^C-butanol blood flow (Fig. 4B).

### Total-Body Parametric Imaging of Blood Flow with ^18^F-FDG

**FIGURE 5.**
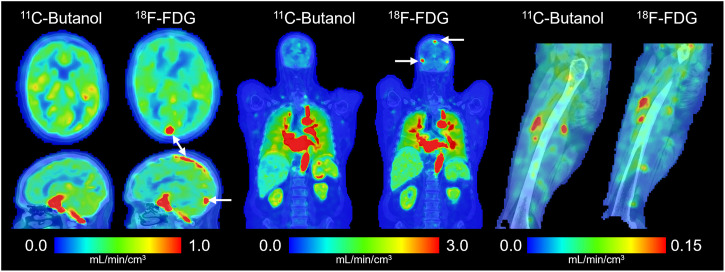
Total-body parametric imaging of blood flow with early dynamic ^18^F-FDG method compared with ^11^C-butanol flow-tracer PET reference in same participant. White arrows indicate sagittal and transverse sinuses in brain.

Total-body parametric images of blood flow generated with ^18^F-FDG and ^11^C-butanol in the same participant are shown in Figure 5. Parametric images appeared similar both visually and in quantitative ranges across the body. A notable difference observed between the 2 blood-flow maps was the absence of sagittal and transverse sinuses in the ^11^C-butanol parametric image. This is likely due to the high extraction fraction of ^11^C-butanol in the brain, resulting in lower venous concentration of the tracer. A representative parametric image of the *T_c_* from a healthy participant is shown in Supplemental Figure 4. One participant had substantial differences in cerebral blood flow between ^11^C-butanol and ^18^F-FDG (Supplemental Fig. 5) (67,68), which may be due to a combination of physiologic and methodologic factors and warrants a future test–retest study.

### Regional ^18^F-FDG Early Kinetics in Healthy Participants

**FIGURE 6.**
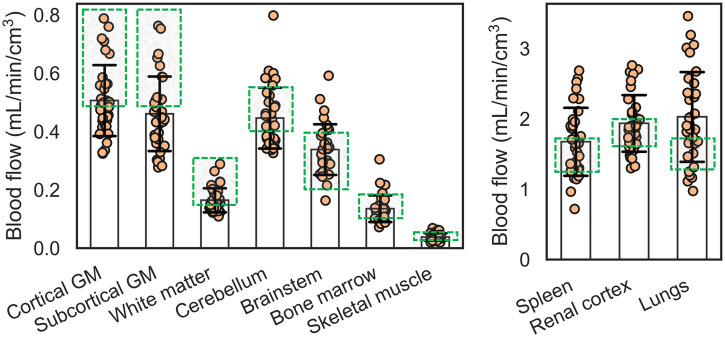
Regional blood flow in 34 healthy participants estimated with early dynamic ^18^F-FDG method. Plots are separated by range of blood-flow values. Average estimates mostly fell within range of average blood-flow values reported in literature (Supplemental Table 1), indicated by green boxes. GM = gray matter.

The distribution of blood-flow estimates with our ^18^F-FDG method in 34 healthy participants is plotted in Figure 6. On average, all tissue regions were within the expected range except the subcortical gray matter and lungs, which were slightly below and above the upper range of average blood-flow values reported in literature, respectively (Fig. 6; Supplemental Table 1) (4). Estimates of *T_c_* (Table 1) reasonably agreed with those reported in the CT/MR perfusion and dual-tracer PET literature (Supplemental Table 1). The identifiability of regional blood-flow estimates with our proposed method was excellent overall across tissue regions (absolute mean error, <5%; SD, <15%) except the skeletal muscle (mean overestimation, 6.4%; Supplemental Table 2).

**TABLE 1:** ^18^F-FDG Early Kinetics Across Healthy Participants with AATH Model (*n* = 34)

Tissue region	Blood flow (mL/min/cm^3^)	*K*_1_ (mL/min/cm^3^)	Extraction fraction	*v_b_* (mL/cm^3^)	*T_c_* (s)
Cortical GM	0.507 ± 0.122	0.136 ± 0.018	0.278 ± 0.046	0.036 ± 0.006	4.4 ± 0.9
White matter	0.165 ± 0.041	0.066 ± 0.009	0.416 ± 0.061	0.018 ± 0.003	6.9 ± 1.4
Subcortical GM	0.461 ± 0.128	0.143 ± 0.019	0.327 ± 0.069	0.033 ± 0.005	4.6 ± 1.5
Brain stem	0.339 ± 0.087	0.125 ± 0.015	0.386 ± 0.082	0.030 ± 0.005	5.6 ± 1.8
Cerebellum	0.447 ± 0.104	0.145 ± 0.016	0.336 ± 0.052	0.037 ± 0.005	5.1 ± 1.0
Spleen	1.676 ± 0.484	1.204 ± 0.404	0.728 ± 0.151	0.166 ± 0.064	6.5 ± 3.3
Renal cortex	1.938 ± 0.402	0.657 ± 0.091	0.348 ± 0.065	0.318 ± 0.039	10.1 ± 1.7
Skeletal muscle	0.039 ± 0.013	0.034 ± 0.012	0.890 ± 0.048	0.017 ± 0.004	29.1 ± 8.3
Bone marrow	0.136 ± 0.046	0.130 ± 0.046	0.954 ± 0.051	0.053 ± 0.018	24.6 ± 9.0
Lungs	2.031 ± 0.639	0.072 (0.059–0.134)*	0.041 (0.033–0.067)*	0.143 ± 0.031	4.4 ± 0.8

* Values are expressed as median with interquartile range in parentheses as a result of 2 measurements shifting distribution (Supplemental Fig. 7).

AATH = adiabatic approximation to tissue homogeneity; GM = gray matter.

Data are expressed as mean ± SD unless otherwise noted.

Regional ^18^F-FDG extraction fraction values in the healthy cohort are summarized in Table 1. The ^18^F-FDG extraction fraction varied greatly among tissue regions across the body. Accordingly, S1TC ^18^F-FDG *K*_1_ was in general agreement with ^11^C-butanol blood flow only in tissues with a high extraction fraction (Supplemental Fig. 6).

## DISCUSSION

We developed an early dynamic ^18^F-FDG PET method for total-body blood-flow imaging with HTR kinetic modeling and validated it against a ^11^C-butanol reference in a subset of participants scanned with both radiotracers. Conventional methods for ^18^F-FDG blood-flow imaging have been limited to tissues with relatively high extraction fraction where blood-to-tissue transport rate *K*_1_ can approximate blood flow. Our proposed method uses a distributed kinetic model (16,17) that explicitly accounts for the blood-flow delivery rate of radiotracer to blood vessels, which was resolved with HTR dynamic imaging. ^18^F-FDG blood-flow estimates were in quantitative agreement with ^11^C-butanol in direct comparisons for the same subjects. We further validated our method with 34 healthy participants, showing that regional blood-flow values across the body were broadly within published ranges (Fig. 6). We report data on ^18^F-FDG extraction fraction values across the body, which varied between tissue types (4%–95%; Table 1). To our knowledge, this is the first study to perform total-body blood-flow imaging with ^18^F-FDG and compare the results with ^11^C-butanol flow-tracer PET in the same subjects.

The key difference between the AATH model and the S1TC model is that the former does not assume that tracer instantaneously mixes in the vascular volume. In the AATH model, blood flow represents the intravascular flow rate through the entire vascular volume residing in a voxel volume. This subtly differs from conventional ^11^C-butanol or ^15^O-water PET measurements of tracer clearance into tissue (*K*_1_), which occurs at the capillaries and is directly proportional to capillary blood flow when *E* approximates 1. However, because our blood-flow estimates were in close agreement with published ranges, it appears that regional blood flow may be tightly coupled with capillary blood flow. This is further supported by the agreement of our AATH regional blood-flow estimates with ranges reported in the literature (Fig. 6). Similarly, AATH *T_c_* represents the mean vascular transit time through the entire vascular volume in a voxel and not only the capillaries. Our *T_c_* estimates generally agreed with those from previously published CT/MR perfusion and dual-tracer PET data (e.g., 3–7 s in the brain) (Supplemental Table 1).

Our data agree with prior work suggesting the need for HTR imaging for distributed kinetic modeling (16–19,25,69). Here, a temporal resolution of 1 to 2 s was required for the AATH model to be preferred over the S1TC model based on the AIC metric (Fig. 3). The temporal resolution may need to be closer to 1 s/frame for tissues such as lung, where the right ventricle input function often has a very fast, sharp bolus (24). Total-body PET now allows the requisite temporal resolution for blood-flow imaging across the body using the widely available ^18^F-FDG radiotracer. Ongoing advancements in image reconstruction may further improve signal-to-noise for HTR imaging (70). Whether these improvements allow lower-dose ^18^F-FDG blood-flow imaging and voxel-level AIC model selection (42) will be considered in future work.

Our method is generally applicable across the body, in contrast to conventional ^18^F-FDG blood-flow estimation methods that require a high extraction fraction. Directly using ^18^F-FDG *K*_1_ as a surrogate of blood flow does not generalize across all tissue types (Fig. 4B). HTR ^18^F-FDG PET has also been used by Larsson et al. (25) to measure cerebral blood flow with model-independent deconvolution analysis, despite the low cerebral extraction fraction of ^18^F-FDG (Table 1). Our estimates of ^18^F-FDG extraction fraction in the cortical gray matter were similar to their estimates in the thalamus (0.19 ± 0.05) as well as those found in another dual-tracer PET study (14). However, our estimates of ^18^F-FDG extraction in other brain regions were larger than these values. Variability in parameter estimates due to differences in estimation methods and modeling has been recognized in MR and CT perfusion (31). The number and characteristics of study participants may have also contributed to this discrepancy. Future work is warranted to compare model-independent deconvolution and AATH parameter estimates and conduct test–retest studies.

Intravenously injected tracers are delivered to tissue vasculature by blood flow, and distributed kinetic models explicitly account for this process (16,18,19). Historically, the AATH model has been used for blood-flow imaging using inert contrast agents and HTR dynamic CT or MRI (30,31). Here, we showed that distributed modeling is applicable to a noninert metabolic radiotracer (^18^F-FDG) as well as a freely diffusible flow radiotracer (^11^C-butanol), provided the requisite temporal resolution is used. Our method may be generally applicable to a wide range of tracers, allowing single-tracer blood-flow imaging. This has been demonstrated in the brain with several PET tracers using the current approach (28) and model-independent deconvolution (25) on HTR dynamic PET, though further validation is required beyond the brain. Numerous opportunities can be enabled for single-tracer multiparametric imaging of physiologic parameters, such as flow-metabolic imaging (1–3) with ^18^F-FDG or joint quantification of blood flow and amyloid burden with amyloid PET tracers (71).

This initial work demonstrated the feasibility of using the AATH model for estimating total-body blood flow with HTR early dynamic ^18^F-FDG PET, but further modeling improvements may be considered at the organ level. First, dual-input modeling with the portal vein is required to enable liver blood-flow measurements (72). However, existing methods (11,72) using ^18^F-FDG *K*_1_ as a surrogate of hepatic blood flow may be sufficient because of the high permeability of liver sinusoids (73). Also, our initial analysis of the myocardium suggested that spillover from the right and left ventricles was substantial at HTR, resulting in substantial blood-flow overestimation. Spillover and cardiac motion correction will be investigated in the future. For the lungs, we used a single right-ventricle input function and neglected the normally small contribution from the bronchial circulation. Dual-input modeling may be required in lung tumors, where the bronchial fraction is nonnegligible (74). The kidneys have complex vasculature, and additional technical considerations, such as partial-volume effect and blood-flow heterogeneity (55), may be required to better model their unique anatomy and vascular transport mechanisms. Lastly, dispersion correction may be required for tissues that are considerably distal to the image-derived arterial input function.

This study had limitations. First, the sample size of participants scanned with both ^18^F-FDG and ^11^C-butanol was small in this pilot study. Instead, the validity of our ^18^F-FDG blood-flow measurements was supported by additional analyses of 34 additional healthy participants. Additional subjects who underwent PET scans with both radiotracers will be analyzed in future studies. Second, participants were not recruited specifically for validation of ^18^F-FDG blood flow. One participant from the dual-tracer group was suspected of having physiologic differences between ^11^C-butanol and ^18^F-FDG scans. Future studies will better account for physiologic confounds by measuring CO_2_ partial pressure, O_2_ partial pressure, and heart rate, among others. Lastly, validation is required in patients with major diagnosed blood-flow defects, such as peripheral, carotid, or coronary artery disease.

## CONCLUSION

We developed an early dynamic ^18^F-FDG PET method with HTR kinetic modeling for total-body blood-flow imaging. Using the ubiquitous ^18^F-FDG radiotracer for blood-flow imaging may mitigate the need for a costly and practically challenging flow-tracer PET scan. In combination with standard ^18^F-FDG PET methods for glucose metabolic imaging, our proposed method may allow efficient single-tracer imaging of blood flow and metabolism, resulting in lower radiation exposure to the patient, shorter scan times, fewer infrastructural requirements, and lower costs. Our method may be generally applicable to other radiotracers, broadening the possibility of single-tracer multiparametric imaging from a single dynamic PET scan.

## DISCLOSURE

The University of California, Davis has a research agreement and a revenue sharing agreement with United Imaging Health Care. This work was supported in part by National Institutes of Health (NIH) grants R01 EB033435 and R61 AT012187. The image data of healthy participants were acquired under the support of NIH R01 CA206187 and P30 CA093373. No other potential conflict of interest relevant to this article was reported.

## Notes

KEY POINTS **QUESTION:** Can HTR early dynamic ^18^F-FDG PET kinetic analysis be used for total-body blood-flow imaging? **PERTINENT FINDINGS:** Blood-flow estimates between ^18^F-FDG and gold standard ^11^C-butanol PET in the same participants showed good quantitative agreement across the body. Regional blood-flow measurements with the proposed early dynamic ^18^F-FDG PET method in 34 healthy participants were within well-established reference ranges in tissues across the body. **IMPLICATIONS FOR PATIENT CARE:** Total-body blood-flow imaging can be performed with the widely available ^18^F-FDG radiotracer, possibly mitigating the need for a dedicated flow radiotracer and expanding opportunities to efficiently study blood flow and glucose metabolism in combination with standard ^18^F-FDG metabolic imaging methods.
